# In Silico Analysis Predicts Nuclear Factors NR2F6 and YAP1 as Mesenchymal Subtype-Specific Therapeutic Targets for Ovarian Cancer Patients

**DOI:** 10.3390/cancers15123155

**Published:** 2023-06-12

**Authors:** Wanja Kassuhn, Pedro R. Cutillas, Mirjana Kessler, Jalid Sehouli, Elena I. Braicu, Nils Blüthgen, Hagen Kulbe

**Affiliations:** 1 Tumorbank Ovarian Cancer Network, 13353 Berlin, Germany; wanja.kassuhn@charite.de (W.K.); jalid.sehouli@charite.de (J.S.);; 2 Department of Gynecology, European Competence Center for Ovarian Cancer, Charité—Universitätsmedizin Berlin, Corporate Member of Freie Universität Berlin, Humboldt-Universität zu Berlin, and Berlin Institute of Health, Campus Virchow Klinikum, 13353 Berlin, Germany; 3 Centre for Haemato-Oncology, Barts Cancer Institute, Queen Mary University of London, Charterhouse Square, London EC1B 6BQ, UK; p.cutillas@qmul.ac.uk; 4 Department of Obstetrics and Gynecology, University Hospital, LMU Munich, Marchioninistr. 15, 81377 Munich, Germany; mirjana.kessler@med.uni-muenchen.de; 5 Department of Obstetrics and Gynecology, Division of Gynecologic Oncology, Stanford University School of Medicine, Stanford, CA 94305, USA; 6 Institute of Pathology, Charité—Universitätsmedizin Berlin, Corporate Member of Freie Universität Berlin, Humboldt-Universität zu Berlin, and Berlin Institute of Health, 10117 Berlin, Germany; nils.bluethgen@charite.de; 7 IRI Life Sciences, Humboldt University, 10117 Berlin, Germany

**Keywords:** ovarian cancer, tumour microenvironment, molecular subtypes, cell–cell communication, therapeutics

## Abstract

**Simple Summary:**

High-grade serous ovarian cancer (HGSOC) is a highly lethal form of ovarian carcinoma characterized by molecular heterogeneity. This heterogeneity is a proposed causal factor in treatment response, development of resistance mechanisms, and increased relapse rates. HGSOC exhibits four molecular subtypes with distinct characteristics. The identification of treatment targets with high efficacy in patient subpopulation such as these molecular subtypes is an intriguing challenge across tumour entities. To exemplarily characterize the mesenchymal subtype of HGSOC and elucidate targeted treatment opportunities, we integrated bulk and single-cell RNA data analysis, and investigated the composition of the TME, its cell–cell communication, and transcription factor regulation. Together with causal inference analysis, we identified several treatment opportunities specific to the mesenchymal subtype. Such targeted treatment based on stratified patient groups may benefit patient survival and highlight the advantages of personalized treatment.

**Abstract:**

Background: Tumour heterogeneity in high-grade serous ovarian cancer (HGSOC) is a proposed cause of acquired resistance to treatment and high rates of relapse. Among the four distinct molecular subtypes of HGSOC, the mesenchymal subtype (MES) has been observed with high frequency in several study cohorts. Moreover, it exhibits aggressive characteristics with poor prognosis. The failure to adequately exploit such subtypes for treatment results in high mortality rates, highlighting the need for effective targeted therapeutic strategies that follow the idea of personalized medicine (PM). Methods: As a proof-of-concept, bulk and single-cell RNA data were used to characterize the distinct composition of the tumour microenvironment (TME), as well as the cell–cell communication and its effects on downstream transcription of MES. Moreover, transcription factor activity contextualized with causal inference analysis identified novel therapeutic targets with potential causal impact on transcription factor dysregulation promoting the malignant phenotype. Findings: Fibroblast and macrophage phenotypes are of utmost importance for the complex intercellular crosstalk of MES. Specifically, tumour-associated macrophages were identified as the source of interleukin 1 beta (IL1B), a signalling molecule with significant impact on downstream transcription in tumour cells. Likewise, signalling molecules tumour necrosis factor (TNF), transforming growth factor beta (TGFB1), and C-X-C motif chemokine 12 (CXCL12) were prominent drivers of downstream gene expression associated with multiple cancer hallmarks. Furthermore, several consistently hyperactivated transcription factors were identified as potential sources for treatment opportunities. Finally, causal inference analysis identified Yes-associated protein 1 (YAP1) and Nuclear Receptor Subfamily 2 Group F Member 6 (NR2F6) as novel therapeutic targets in MES, verified in an independent dataset. Interpretation: By utilizing a sophisticated bioinformatics approach, several candidates for treatment opportunities, including YAP1 and NR2F6 were identified. These candidates represent signalling regulators within the cellular network of the MES. Hence, further studies to confirm these candidates as potential targeted therapies in PM are warranted.

## 1. Introduction

Due to its asymptomatic characteristics and heterogeneous nature, epithelial ovarian cancer (EOC) remains a challenging disease. High-grade serous ovarian cancer (HGSOC) is the most common histological type accounting for the majority of gynaecological cancer related death (https://www.cdc.gov/cancer/ovarian/statistics/index.htm; assessed on 20 March 2021) [1,2]. Despite some success with the introduction of poly-ADP ribose polymerase (PARP) inhibitors in response to mutations in breast cancer type 1 and 2 susceptibility protein (BRCA1/2), there has been little improvement in the prognosis over the past two decades [3]. Indeed, around 80% of HGSOC patients develop recurrent disease, despite surgical intervention and complete response to first-line chemotherapy [4].

Previous landmark studies by the Australian Ovarian Cancer Study (AOCS) [5] and The Cancer Genome Atlas (TCGA) [6] identified four molecular subtypes termed mesenchymal (MES), immunoreactive (IMR), differentiated (DIF), and proliferative (PRO). These subtypes exhibit distinct gene expression profiles indicative of diverse tumour biology with consequences on clinical characteristics such as chemoresistance and patient outcome [7,8].

However, the underlying biological mechanisms and key regulators driving these molecular subtypes are not sufficiently recognized to be implemented in clinical management. In fact, patient stratification failed to demonstrate substantial differences in response rate to select treatment strategies [9]. Furthermore, Schwede et al. [10] recently questioned the reliability of HGSOC subtype classification algorithms and their reliance on genes expressed exclusively in the stromal compartment. Thus, the stroma admixture or tumour purity could prevent accurate subtyping. Due to this circumstance, the predominance of MES, as well as its invasive characteristics and clinical presentation, we characterized its tumour microenvironment (TME), intercellular signalling, and gene regulatory mechanisms in this study. Thus, we aim to identify novel therapeutic targets for personalized treatment of MES.

Cytokines are key mediators of cell communication in the TME. We have previously described a dynamic inflammatory cytokine network driven by interleukin-6 (IL6), tumour necrosis factor (TNF), and C-X-C motif chemokine ligand 12 (CXCL12) in human ovarian cancer cell lines, patient biopsies, and murine xenografts [11]. These crucial cytokines were found to mediate cancer-related inflammation and promote a malignant phenotype observed in ovarian cancer. Similarly, complex cytokine networks were also reported in multiple other cancer entities [12,13,14]. Aberrant production of inflammatory cytokines is a common consequence of oncogenic change. Thus, malignant transformation can influence the TME, particularly via the production of pro-tumoral cytokines, which is reflected in a gene expression signature linked to the cytokine network and associated with angiogenesis, cell adhesion, cell cycle, and inflammatory signalling [11].

Currently, only few ovarian cancer single-cell RNA sequencing (scRNA-seq) datasets have been published, and even fewer provide the necessary basis for cell-level resolution investigation of the TME and cellular communication. Recently, however, Olbrecht et al. [9] published a uniquely suited scRNA-seq dataset including subtype classifications of corresponding bulk RNA sequencing (RNA-seq) data. Nevertheless, the technical limitations and biological constraints accompanying scRNA-seq including small sample sizes, noise, and higher complexity compared to bulk data are evident and raise computational challenges [15]. To alleviate some of these limitations, Bao et al. [16] integrated bulk and single-cell data to investigate tumour heterogeneity in triple-negative breast cancer. Moreover, many useful algorithms have been developed exclusively for bulk data. For example, the CARNIVAL [17] algorithm was developed to predict potential upstream alterations that drive expression changes and thus provide insights into disease.

Here, we integrated both bulk and single-cell RNA data driven analysis to reconstruct the subtype-specific heterogeneity of the TME. To further leverage the superior resolution of scRNA-seq data, the cell–cell communication driving malignant transcriptional programs in MES was also investigated. Finally, a causal inference framework was used to identify novel therapeutic targets.

## 2. Methods

### 2.1. Gene Expression Profiles and Data Pre-Processing

Publicly available gene expression profiles of HGSOC patients from Tothill et al. [5] (GSE9891) and TCGA [6] (GSE82191) were downloaded from the Gene Expression Omnibus (GEO, https://www.ncbi.nlm.nih.gov/geo/; assessed on 16 July 2019) and TCGA (https://portal.gdc.cancer.gov/; assessed on 21 February 2021). Following normalization with robust multi-array average (RMA), intensities of probes were collapsed utilizing the WCGNA [18] R package and a ‘MaxMean’ setting.

Subsequently, the consensusOV [19] R package was used to classify all samples by HGSOC subtypes. To generate ideal subtype representations, samples from the Tothill dataset with congruent predictions by all classifiers integrated in consensusOV (*n* = 119) were selected for further analysis.

For validation, independent gene expression profiles from TCGA were similarly pre-processed and filtered for high-grade serous histological type (*n* = 475). Again, subtypes were determined via consensusOV. However, to investigate the reproducibility of our analysis on heterogeneous samples or data with increased biological variation, no selection was performed on the validation set.

To visualize the interrelationship between samples and assess a general goodness of the subtype classifications, a Uniform Manifold Approximation and Projection (UMAP) embedding was calculated based on either the first 12 or 10 PCs (Tothill and TCGA, respectively).

### 2.2. Enrichment of Hallmark Gene Signatures

To characterize the HGSOC subpopulations, we performed a cancer hallmark enrichment analysis. To that end, the hallmark gene sets (v7.0; *n* = 50) were obtained from MSigDB [20] (Molecular Signatures Database) and evaluated with fast gene set enrichment analysis (fgsea).

### 2.3. In Silico Enrichment of Immune and Stromal Cell Type Signatures

We applied xCell [21] to investigate the heterogeneity of tumour microenvironment with respect to HGSOC subtypes. xCell performs single-sample GSEA utilizing 489 immune and stromal cell phenotype specific gene sets. To allow for comparison of compositional distinctions of the tumour microenvironment between subtypes, NESs of each cell type were averaged by subtypes and scaled.

### 2.4. Complementary Single-Cell Analysis of Tumour Composition

To further investigate compositional differences at a single-cell resolution, we obtained scRNA-seq data of HGSOC patients (*n* = 4) from Olbrecht et al. [9]. Molecular subtypes of these samples (*n* = 4; one per subtype) were generated from corresponding bulk sequencing data via the consensusOV algorithm. In total, 5306 cells with cell type annotation were obtained (doublets removed).

To estimate the proportion of the epithelial, stromal, and immune compartment of HGSOC subtypes, the cells of each sample were separately normalized and scored based on an epithelial, stromal, and immune gene signature as described by Smillie et al. [22]. Subsequently, the cell-level scores of each signature were z-score normalized and cells were assigned to either an epithelial, stromal, or immune compartment based on the highest score.

### 2.5. Cell–Cell Communication Analysis Combined with Cytokine Activity Inference

To further investigate cell–cell communication in the distinct TME of mesenchymal HGSOC, we applied the ligand–receptor analysis framework LIANA [23]. To that end, the data of the mesenchymal sample was initially filtered and log_2_-transformed. Moreover, all subclusters comprising fewer than 5 cells were removed. Next, the analysis was performed on the *consensus* ligand–receptor interactions with 1000 iterations. The resulting interactions were subsequently filtered by the aggregated ranks (rank < 0.05).

Next, we applied the NicheNet [24] algorithm to estimate ligand activity based on downstream gene expression in tumour cells of MES. Concordant with our bulk GSEA analysis and likely to be affected by ligands, we evaluated a set of cancer hallmarks that were enriched in MES, including *epithelial–mesenchymal transition*, *inflammatory response*, *TNF-α signalling* via *NF-κB*, *TGF-β signalling*, and *angiogenesis*.

To investigate the influence of the TME on the tumour cells, all ligands involved in ligand–receptor interactions directed at tumour cells were considered for the NicheNet analysis, applying 5 rounds of 5-fold cross-validation. Lastly, we calculated the regulatory potential between the top LIANA-prioritized ligands (*n* = 10) and their target genes from the hallmark signatures. To that end, ligands were removed from the hallmark gene signatures.

### 2.6. Correlation of Ligand–Receptor Pair Expression with Cancer Hallmarks

To investigate the extent to which the ligand–receptor pairs identified by LIANA were correlated with the cancer hallmarks interrogated in our NicheNet analysis, we performed ssGSEA of cancer hallmarks on the MES subtype samples of the Tothill dataset (*n* = 42). Subsequently, we calculated Pearson correlation coefficients between the average expression of ligand–receptor pairs and the enrichment scores of each sample.

### 2.7. Inference of Transcription Factor Activity from Gene Expression Data

To evaluate master regulators that drive the distinct transcriptional programming in the MES subtype, gene expression profiles of HGSOC subtypes were analysed with the Virtual Inference of Protein-activity by Enriched Regulon analysis (VIPER) [25] algorithm. To that end, DoRothEA, a gene regulatory network providing transcription factor–target interactions, was utilized as reference (confidence level C and above) [25,26]. Only transcription factors with at least ten target genes were considered (*n* = 289; interactions, *n* = 31,539).

### 2.8. Inferred Transcription Factor Activity-Guided Intercellular Communication Network

To construct an intercellular communication network focused on transcription factors, ligand–receptor–transcription factor interactions and transcription factor–cytokine interactions were downloaded from CellCall [27] and CytReg (https://cytreg.bu.edu/about.html; assessed on 21 August 2019) resources, respectively. LIANA-identified ligand–receptor pairs affecting gene regulation in tumour cells were linked to transcription factors based on CellCall’s interactions. Here, 29 of the transcription factors with the highest absolute NES (*n* = 50) as calculated by VIPER were retained based on the availability of downstream regulatory interactions in the CytReg resource. Only human directed transcription factor-cytokine interactions from CytReg identified by functional assay with known mode of action (*n* = 109) were incorporated. Subsequently, we used the limma tool to measure regulatory effects as differential expression of cytokines (log_2_FC). The complexity of cytokine expression was represented by the number of dysregulated transcription factors regulating the cytokine.

### 2.9. Generation of a Prior Knowledge Network

The VIPER analysis lacks information about the topology of signalling pathways. Utilizing a prior knowledge network such as OmniPath [28], upstream regulators can be inferred from downstream signalling targets [28]. Human protein interactions were downloaded from OmniPath. Only directed and signed interactions were included, if confirmed by at least three independent sources (e.g., curated databases). Interactions annotated as both stimulating and inhibiting were split into two individual interactions, each with a single mode of regulation.

### 2.10. Contextualization of Signalling Networks via Causal Reasoning

To identify the principal driver(s) of the mesenchymal HGSOC transcriptional network, the causal inference algorithm CARNIVAL [17] was applied. CARNIVAL integrates VIPER transcription factor activities, PROGENy [29] pathway activities, the prior knowledge network and a list of known or potential targets of perturbation to infer the cause of downstream transcription factor activities [17].

Here, the top transcription factors (*n* = 100) ranked by absolute NES were evaluated. A β-weight of 0.9 was applied for high reproducibility due to strict node penalty. The UniProtKB (https://www.uniprot.org/help/uniprotkb; assessed on 6 October 2020) resource was queried using the search terms: ‘*keyword: “Kinase [KW-0418]” AND reviewed: yes AND organism: “Homo sapiens (Human) [9606]”*’ to extract a list of kinases (*n* = 637) that served as potential targets.

To identify novel therapeutic targets, we first performed CARNIVAL analysis comparing the MES subtype against the combined other subtypes. Subsequently, we repeated this analysis in a pairwise manner. For the pairwise approach, we additionally performed the reciprocal comparisons and excluded inconsistent results. The targets were identified by the intersection of all pairwise results and computationally validated on the TCGA dataset.

## 3. Results

### 3.1. Classification and Characterization of High-Grade Serous Ovarian Cancer Datasets

To characterize the MES subtype and identify novel therapeutic targets, a dataset comprising ideal representations of HGSOC subtypes was generated by excluding samples with inconsistent subtype classifications across several tools, likely due to heterogeneity. First, the publicly available dataset from Tothill et al. (GSE9891) [5] was obtained and expression profiles were filtered for histological type of HGSOC, normalized, and consensus classified with consensusOV. Subsequently, the Tothill samples were filtered for ideal representations of HGSOC subtype characteristics (*n* = 119). This resulted in 42 (35.3%) MES samples, and 33 (27.7%), 16 (13.4%), and 28 (23.5%) samples of the IMR, DIF, and PRO subtypes, respectively. For validation, expression profiles from TCGA (GSE82191) [6] were utilized. In contrast to the Tothill dataset, all samples of the HGSOC histological type (*n* = 475) were retained. Here, classification resulted in 105 (22.1%), 139 (29.3%), 140 (29.5%), and 91 (19.2%) samples of the MES, IMR, DIF, and PRO subtypes, respectively.

**Figure 1:**
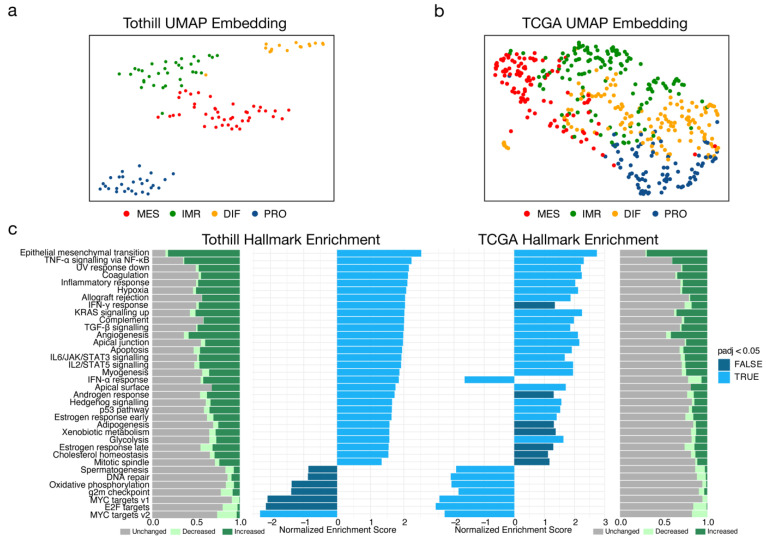
**Characterization of molecular subtypes of high-grade serous ovarian cancer (HGSOC) in two independent datasets.** (**a**) Uniform Manifold Approximation and Projection (UMAP) non-linear dimensional reduction analysis that visualizes the relationships between samples stratified by HGSOC subtype from Tothill (*n* = 119; GSE9891). Samples were filtered to best describe an ideal representation of HGSOC subtypes by selecting only samples with consistent predictions across subtyping tools integrated in consensusOV. (**b**) UMAP analysis of the TCGA (*n* = 475; GSE82191) dataset. (**c**) Enrichment of cancer hallmarks for mesenchymal tumours of the Tothill and TCGA datasets. Individual expression of genes from hallmark signatures were arbitrarily categorized as decreased (log_2_ FC < −0.25), unchanged (−0.25 ≤ log_2_ FC < 0.25), increased (log_2_ FC ≤ 0.25), or displayed as bar plots.

To evaluate whether the filtering procedure resulted in stronger segregation between subtypes, UMAP representations of both datasets were generated (Figure 1a,b). The UMAP of the filtered data showed clustering of samples of the same subtype suggesting stronger similarity. On the other hand, the unfiltered TCGA data showed an increased mixture of samples of different subtypes indicative of less accurate classification. Thus, characterization and comparison between samples of different subtypes may be more accurate after filtering.

GSEA of cancer hallmarks in the previously classified subpopulations enabled further comparison of datasets with respect to malignant programs. The MES subtype was characterized by a drastic increase in the epithelial mesenchymal transition (EMT) program compared to the other subtypes (Figure 1c). Congruently, signalling pathways mediated by the pro-inflammatory cytokines TNF and transforming growth factor beta (TGFB1) showed significant enrichment; both are known to be strong inducers of EMT [30]. Furthermore, the JAK/STAT signalling pathway mediated by signal transducer interleukin-6 (IL6) was significantly enriched. In general, expression profiles of the MES subtype were characterized by upregulation of genes linked to processes such as angiogenesis, hypoxia, inflammation, and EMT. With the exception of the IFN-α response hallmark, these results were largely reflected in the TCGA data (Figure 1c). Hence, the filtered dataset reflected the characteristics of MES and could be used for precise characterization of MES.

### 3.2. Compositional Analysis of High-Grade Serous Ovarian Cancer Subtypes Reveals Distinct Cell Type Proportions

To investigate and characterize the cell type composition within HGSOC subtypes, the xCell [21] algorithm was applied to the filtered dataset. As a crucial driver of malignant phenotypes in tumours, important for both pro-tumoral and anti-tumoral processes, the cellular heterogeneity of the TME is of utmost importance [21]. Hence, cell type-specific gene signatures were tested for enrichment to allow for the dissection of cellular heterogeneity.

**Figure 2:**
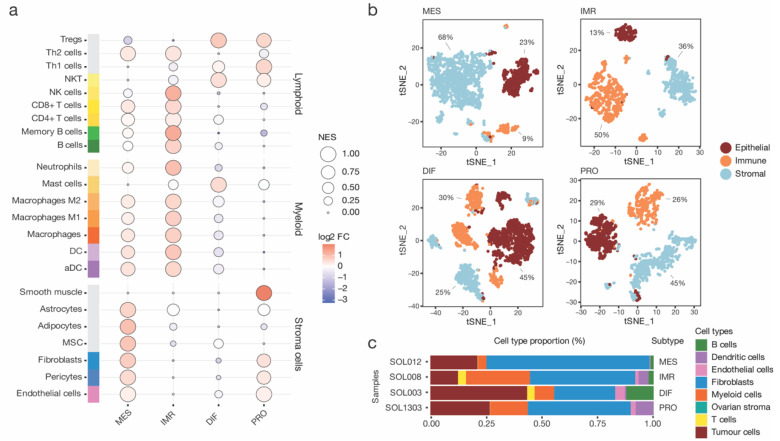
**Compositional analysis of high-grade serous ovarian cancer (HGSOC) in bulk and single-cell RNA sequencing data.** (**a**) Outcome of a xCell analysis. Samples of each HGSOC subtypes were analysed by single-sample Gene Set Enrichment Analysis (GSEA). Cell type-specific enrichment scores were averaged by tumours of mesenchymal (MES), immunoreactive (IMR), differentiated (DIF), and proliferative (PRO) subtype, and scaled for visualization. Importantly, enrichment scores are not equal to the proportion of cell types. In addition, the log_2_ fold change (log_2_ FC) to the dataset mean was calculated. (**b**) Four single-cell RNA sequencing samples of HGSOC patients corresponding to each subtype were obtained. All cells were scored for an epithelial, immune, and stromal gene signature and assigned to one of the three cell compartments. Moreover, the cell proportions of each compartment were annotated. (**c**) Compositional analysis of each subtype based on annotated cell types.

In general, the mesenchymal subtype was dominated by a high influx of stromal cells, including mesenchymal stem cells, adipocytes, and fibroblasts (Figure 2a). These cell phenotypes have been known to be crucial components of the mesenchyme and contribute to tumour pathogenesis [31]. In contrast to the IMR subtype that exhibited enrichment of several myeloid and lymphoid cell phenotypes, immune cell infiltration in MES was notably lacking. Additionally, dendritic cells, monocytes, and macrophage phenotypes were enriched in the IMR subtype compared to the DIF and PRO subtypes.

While bulk analysis with xCell could rely on a higher number of samples to reduce sample-specific biases, it lacks cell-level resolution. To alleviate this limitation, further interrogation of the HGSOC scRNA-seq data published by Olbrecht et al. [9] enabled more extensive investigation the TME composition. To that end, cells of samples corresponding to each subtype were individually scored utilizing an epithelial, stromal, and immune cell signature as described by Smillie et al. [22] (Figure 2b). Next, the scores were z-transformed and used to assign each cell to one of the three compartments. The MES subtype comprised 68% stromal cells, 23% epithelial cells, and 23% immune cells (Figure 2b). In congruence with the bulk xCell results, the MES stromal compartment was composed of a majority of fibroblasts and few endothelial cells (Figure 2c). The epithelial and immune compartment consisted mainly of tumour cells and myeloid-derived cells, respectively. In contrast, the IMR, DIF, and PRO subtypes exhibited distinct cell compartment proportions, with significantly fewer stromal cells. For example, the IMR subtype sample was composed of 50% immune cells, including myeloid cells and T-cell and B cell phenotypes. These results suggest a high importance of fibroblasts and macrophage phenotypes for tumour cells to establish the malignant milieu observed in MES.

### 3.3. Cell–Cell Interactions Drive Cancer Hallmarks in High-Grade Serous Ovarian Cancer

To investigate the cell–cell communication facilitated by ligand–receptor interaction between these cell types, the recently published LIANA tool was applied. LIANA is a wrapper for several tools that aims to identify ligand–receptor interactions between cell types in scRNA-seq data. Here, LIANA parsed the log_2_-normalized HGSOC scRNA-seq data of MES sample to identify ligand–receptor interactions between 13 cell phenotypes as previously annotated [9]. These included B cells (BC_IGHG1_PRDM1), fibroblasts (FB_CALB2, FB_CFD, FB_COL27A1, FB_COMP, FB_MYH11, FB_RGS5, FB_SERPINE1), myeloid cells (M_CCL18, M_CCR2, M_CD14), and tumour cells (Tum_KRT6A, Tum_TNNT2).

**Figure 3:**
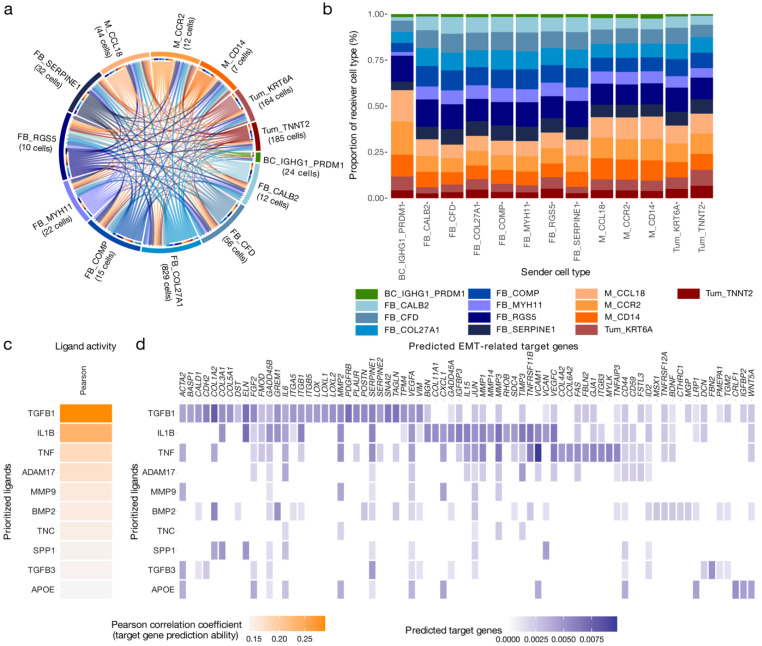
Cell–cell communication and effects on downstream gene expression in tumour cells of mesenchymal high-grade serous ovarian cancer. (**a**) Ligand–receptor interactions between 13 cell type phenotypes of the mesenchymal subtype (MES); cell type subclusters with less than five cells were excluded from the analysis. In total, LIANA, a ligand–receptor analysis framework, identified 21,565 significant interactions based on the aggregated rank (AR < 0.05) between several tools that aim to identify ligand–receptor interactions. (**b**) To characterize sender cell types based on the proportion of interactions, the reported interactions were stratified by sender cell type and juxtaposed for comparison. (**c**) Results of the NicheNet ligand activity prediction on the epithelial–mesenchymal transition (EMT) cancer hallmark gene set; the 10 ligands that best predicted target gene expression, evaluated by Pearson correlation coefficient. Only LIANA prioritized ligands involved in interactions directed at tumour cell subclusters and available in the NicheNet resource were considered (*n* = 109). Ligands were removed from the hallmark gene set prior to analysis. (**d**) NicheNet’s ligand–target matrix depicting the regulatory potential between ligands and EMT genes.

LIANA analysis retained a total of 21,565 significant ligand–receptor interactions in MES (Figure 3a). The highest number of interactions were reported to involve fibroblasts (*n* = 18,123), specifically the FB_RGS5 subcluster that was defined as pericytes (*n* = 4678). In contrast, B cells were characterized by fewest interactions (*n* = 667) (Table S1). To check if this was an artifact stemming from the number of subcluster of major cell types, the number of unique interactions of each major cell type was determined. However, even after removing duplicate interactions, of the remaining interactions (*n* = 3318), fibroblasts retained the most (*n* = 2361) and B cells the fewest (*n* = 319).

The LIANA-identified interactions were composed of 439 unique ligands and 480 unique receptor complexes. The most frequently interacting ligands included fibronectin1 (FN1), collagen type I alpha 1 chain (COL1A1), TGFB1, G protein subunit alpha I2 (GNAI2), and collagen type I alpha 2 chain (COL1A2). CXCL12, also known as the stroma cell-derived factor 1, was ranked 21st out of 439. Several members of the fibroblast growth factor (FGF) family, including FGF1, FGF7, FGF9, FGF13, and FGF18, were also identified as factors predicted to be secreted nearly exclusively by the fibroblast population. However, FGF18-mediated interactions also originated in both tumour cell subclusters.

To further investigate auto- and paracrine interactions directed at cancer cells, interactions targeting tumour cell subclusters (*n* = 1991) mediated by 189 unique ligands were selected for additional analysis. Notably, these interactions were characterized by secreted phosphoprotein SPP1, TGFB, and mediators of canonical Wnt signalling pathways. Specifically, SPP1-ITGB1 was previously shown to promote progression in ovarian cancer via the ITGB1/FAK/AKT signalling pathway [32]. Interestingly, Wnt Family Member 7A (WNT7A) was only secreted by tumour cells themselves.

IL1B was exclusively sourced from myeloid cells, specifically the M_CCL18 subcluster previously described as tumour-associated macrophages (TAMs) [9]. In addition, myeloid subclusters were the main source of interactions involving members of the tumour necrosis factor superfamily, including TNFSF12-TNFRSF12A and TNFSF13B-TFRC.

Next, sender cell types were characterized by the proportion of receiver cell types (Figure 3b). Most notable, interactions sourced from B cells comprised a distinctly larger proportion of interactions targeting monocyte subclusters. Moreover, receiver cell type proportions fluctuated across fibroblast subclusters but were highly similar amongst myeloid cell subclusters. In general, tumour cell subclusters were less frequently targeted by interactions than other cell types in the TME (Table S1).

### 3.4. IL1B, TGFB1 and TNF Are Key Drivers of the Cancer Hallmarks in the MES Subtype

To extend on the ligand–receptor analysis and by exploring the effects of LIANA-identified ligands on downstream gene expression in tumour cells, a NicheNet analysis was implemented. NicheNet utilizes a prior model based on existing knowledge on ligand–target regulatory potential to infer ligand activity in the receiver niche [24]. Here, a list of LIANA-prioritized ligands involved in interactions directed at tumour subclusters and scRNA-seq data of tumour cell of MES were analysed with NicheNet. In detail, ligand activity was evaluated in the context of cancer hallmarks enriched in MES as determined by bulk GSEA analysis (Figure 1c). Of the 189 unique ligands, only 109 were available in the NicheNet reference resources and consequently analysed.

IL1B, TGFB1, TNF, and other ligands were identified as key drivers of downstream gene expression associated with the EMT hallmark in MES (Figure 3c). Next, the potential regulation of EMT genes was assessed, with many genes involved in EMT predicted to be regulated by the identified ligands (Figure 3d). In fact, 35.4% of the EMT hallmark genes were among the 5% of most strongly predicted target genes, compared to 3.6% of non-EMT hallmark genes (Fisher’s exact test: 7.5 × 10^−7^) (Table S2). This suggests that MES undergoes IL1B-, TGFB1-, and TNF-induced EMT, with each cytokine having regulatory effects on a subset of genes linked to EMT.

This analysis was repeated with four additional hallmarks linked to the MES subtype (Figure S1). Again, the primary ligands predicted to drive hallmark programs in MES were identified. For example, the ligand strongest predicting inflammatory response hallmark genes was TNF. TNF is well known as a pro-inflammatory cytokine involved in complex signalling networks that drive ovarian cancer [11]. The ten ligands with the highest activity with respect to inflammatory response genes were able to predict 31.4% of hallmark genes among the 5% of most strongly predicted target genes, compared to 3.7% of non-hallmark genes (Fisher’s exact test: 4.2 × 10^−34^) (Table S2).

To further investigate the link between ligand–receptor pairs and cancer hallmarks, ssGSEA of cancer hallmarks was performed on the bulk MES samples of the Tothill dataset (*n* = 42). Next, the enrichment scores of each sample were correlated with the average expression of the ligand–receptor pairs.

The expression of several ligand–receptor pairs was highly correlated with the enrichment scores of the aforementioned hallmarks (Figure S2a) (Table S3). Moreover, there were overlaps of highly correlated ligand–receptor pairs between hallmarks including those previously identified to be highly predictive for hallmark expression from our previous analysis (Figure 1c). In fact, expression of the ligand–receptor pair IL1B–IL1R1 exhibited high correlation with enrichment scores of several of the investigated hallmarks (Figure S2b). In addition, several of the ligands identified by NicheNet were also significantly overexpressed in MES compared to other subtypes (Figure S2c). The most prominent differentially expressed ligand across subtypes was secreted frizzled-related protein 2 (SFRP2), which is involved in the induction of Wnt signalling.

This analysis suggested strong involvement of IL1B, TGFB1, and TNF in several cancer hallmarks identified in both bulk and single-cell data of MES.

### 3.5. Intercellular Signalling Mediates Dysregulation of Transcription Factor Activity and Aberrant Cytokine Production in MES

**Figure 4:**
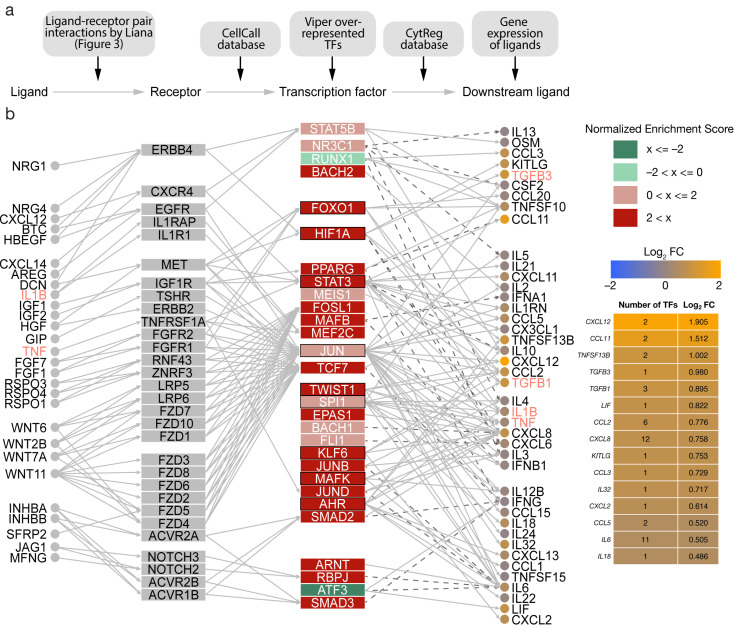
Transcription factor-guided intercellular communication network in the mesenchymal subtype (MES) of high-grade serous ovarian cancer (HGSOC). (**a**) An overview of resources and data used to generate the communication network. (**b**) The 29 most dysregulated transcription factors with available regulatory interactions were utilized as basis to generate a regulatory network between ligand–receptor pairs, transcription factors, and downstream signalling molecules. LIANA-identified ligand–receptor pairs affecting gene regulation in tumour cells were linked to transcription factors using the CellCall resource. Human transcription factor–cytokine interactions identified by functional assay were gathered from the CytReg resource. Outlines represent transcription factors with regulatory interactions with EMT ligands identified by NicheNet. Solid edges describe stimulating interactions while dashed edges depict inhibition. Cytokine importance for the MES subtype was estimated via the differential gene expression of cytokines measured in log_2_ fold change (log_2_ FC) between MES and the other subtypes of HGSOC. Moreover, the complexity of cytokine transcriptional regulation was highlighted by the number of transcription factors interacting with each cytokine.

To further investigate the effects of IL1B, TGFB1, TNF, and other identified ligands on downstream transcription factor activity and in turn expression of signalling molecules, transcription factor activity estimates were combined with the CellCall and CytReg (https://cytreg.bu.edu/about.html; assessed on 21 August 2019) resources (Figure 4a). The CellCall and CytReg resources comprise ligand–receptor–transcription factor and transcription factor–cytokine regulatory interactions, respectively. First, the VIPER tool facilitating inference of transcription factor activity based on the expression of direct target genes was used to calculate the normalized enrichment of transcription factors (*n* = 289) as a proxy for their activity (Table S4).

Notably, the erythroblast transformation-specific (ETS) transcription factor ERG displayed the highest enrichment (NES = 3.031) based on 88 target genes. VIPER also identified SMAD2/3 amongst the top 20 dysregulated transcription factors (NES = 2.550 and NES = 2.744, respectively). SMAD2/3 have been linked to the promotion of EMT in ovarian cancer involving the TGFB pathway [33]. Likewise, the major regulator of angiogenesis hypoxia inducible factor 1 alpha (HIF1A) (NES = 2.448) and Twist-related protein 1 (TWIST1) (NES = 2.792), a downstream target of the HIF1A, were also upregulated. Both HIF1A and TWIST1 are integral to the PI3K pathway, which was described to be crucially involved in the tumorigenesis of ovarian cancer [34]. Moreover, HIF1A and TWIST1 are involved in TGFB-induced activation of EMT [30]. Generally, the activity of related transcription factors was increased, including STAT3/5, members of the STAT family of transcription activators, TEAD1/4, FOSL1/2, and JUN. RUNX1 and ATF3 had negative enrichments (NES = −1.833 and NES = −2.459, respectively) indicating less activity (Figure 4b). Results of the complete analysis have been provided (Table S4).

Next, a network connecting the LIANA-identified ligand–receptor pairs, the most dysregulated transcription factors (*n* = 29), and downstream cytokines was generated based on directed interactions. In addition, log_2_FC were integrated to evaluate regulatory effects.

Several signalling axes characterized by ligand-driven receptor activation, downstream signalling cascades, and transcription factor dysregulation were identified in MES. In detail, activated transcription factors, such as FOXO1, HIF1A, STAT3, and JUN, regulate the expression of TGFB1, TGFB3, IL1B, and TNF, promoting cancer hallmarks associated with these ligands.

Moreover, IL6 was the second highest connected cytokine in the network (Figure 4b). However, it was not significantly overexpressed compared to the other subtypes (log_2_FC = 0.505; *p* = 0.067). Increased activity of STAT3, TWIST1, SPI1, JUN, JUND, ARNT, and RBPJ mediate the expression of IL-6 via MAPK, PI3K, and JAK/STAT pathways. Interestingly, ATF3, which acts as an inhibitor of IL-6, was downregulated. In contrast, several other interleukins were significantly overexpressed, including the leukaemia inhibitory factor LIF (log_2_FC = 0.822; *p* = 1.7 × 10^−0.5^), CXCL8 (log_2_FC = 0.758; *p* = 0.049), and IL32 (log_2_FC = 0.717; *p* = 0.019). Consistent with the cancer hallmark analysis, TNF (TNFSF13B), TGFB1, and TGFB3 played important roles in the mesenchymal microenvironment crosstalk. Furthermore, the chemokines CXCL12, CCL11, CCL2, CCL3, CCL5, and CXCL2 were differentially expressed.

### 3.6. Causal Inference Analysis Reveals YAP1 and NR2F6 as Novel Therapeutic Targets

To identify novel therapeutic targets that interrupt the upstream signalling cascades between transcription factors and receptors, we performed a causal inference analysis with CARNIVAL. The CARNIVAL pipeline aims to elucidate targets causally involved in driving the regulatory cascades and thus transcription factor dysregulation. Here, the inferred transcription factor activities in MES were utilized to identify upstream kinases and intermediate nodes according to a prior knowledge network from OmniPath.

**Figure 5:**
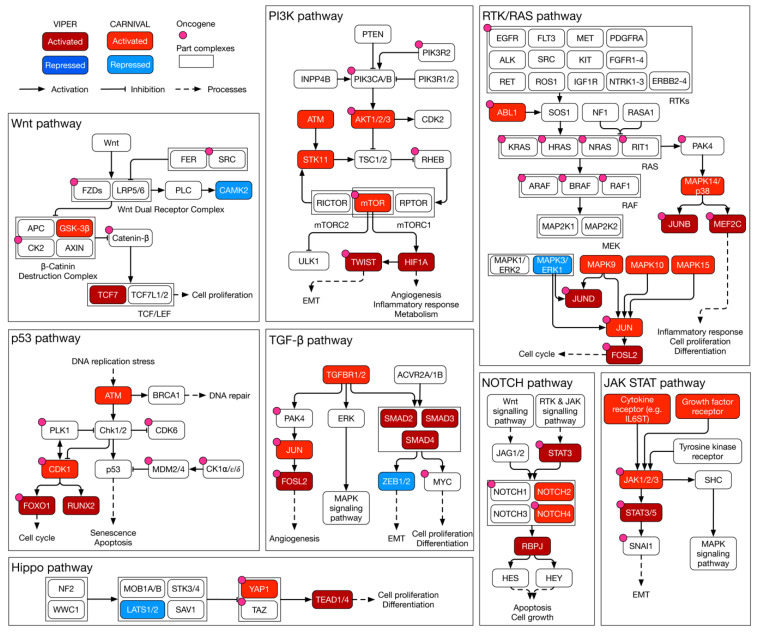
**Schematic summary of inferred kinase signalling circuitry within key signalling pathways of MES in HGSOC.** A curated overview of key signalling pathways in MES of HGSOC. Within each pathway, transcription factor activities as estimated by VIPER are highlighted. Likewise, the modes of activity of CARNIVAL-inferred causal upstream kinases are included. RTK: receptor tyrosine kinase; EMT: epithelial–mesenchymal transition.

CARNIVAL predicted IL-6ST, EGFR, KIT, MPL, and CCR5 to activate the JAK/STAT signalling cascade (Figure 5). In response, JAKs modulate TWIST1 activity via STAT3 stimulation. Moreover, multiple members of the MAPK family (MAPK9, MAPK10, MAPK14, and MAPK15) were predicted to activate JUN and in turn FOSL2. Furthermore, large tumour suppressor kinase 1 (LATS1) was necessary for the activation the YAP1 and transcriptional enhanced associate domain (TEAD) transcription factor complex. LATS1, YAP1, and the TEAD transcription factor family are members of the HIPPO pathway promoting cell survival, proliferation, and survival [35]. A schematic summary of the findings within key signalling pathways is shown in Figure 5.

**Figure 6:**
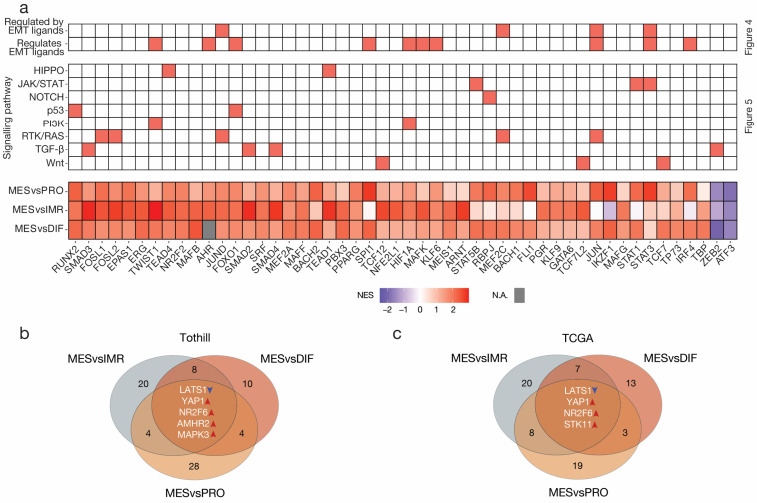
**Pairwise transcription factor dysregulation and causal inference analysis in high-grade serous ovarian cancer.** (**a**) Heatmap summarizing the outcome of regulatory network analysis, causal inference analysis, and the pairwise VIPER transcription factor activity inference analysis; the highest ranked transcription factors (*n* = 50) according to absolute normalized enrichment score (NES) across the three comparisons. Missing values are displayed in grey. The intersection of nodes in the CARNIVAL causal inference network in the (**b**) Tothill and (**c**) TCGA datasets. Targets were identified from the three-way intersection of all pairwise comparisons. To limit erroneous results, all pairwise analyses were performed both ways and contradictory nodes excluded.

However, this global approach disregarded the biological variance between subtypes in the reference group. To alleviate this shortcoming, this analysis was repeated in a pairwise manner. Again, VIPER analysis first inferred transcription factor activities. Like the previous VIPER analysis, consistently hyperactivated transcription factors, including FOSL2, JUND, NR2F2, RUNX2, and TEAD4 were amongst the most dysregulated transcription factors (Figure 6a, Table S4).

Next, pairwise causal inference analysis was performed. To identify targets, we calculated the three-way intersection of nodes of all three pairwise CARNIVAL output networks excluding the downstream transcription factors themselves. The intersection revealed five targets with consistent modes of activity in the MES subtype compared to all other subtypes (Figure 6b). Remarkably, the analysis on the validation dataset reproduced three of these targets, namely LATS1 suppression, and YAP1 and NR2F6 activation (Figure 6c). In addition, activation of STK11 was observed in the TCGA analysis. On the other hand, the Tothill analysis revealed AMHR2 and MAPK3 as consistently enriched in MES. This stands in contrast to the global analysis that inferred MAPK3 to be repressed. Re-evaluation of these results showed that downstream transcription factors of MAPK3 including JUN, JUND, TWIST1, HIF1A, and STAT3 were in fact activated. This could be an erroneous result based on pathway weighting in the global analysis.

## 4. Discussion

In our complementary bulk RNA and scRNA-seq study we leveraged the advantages of both technologies and applied a range of specialized tools to characterize the TME, the cell–cell communication, and its effects on transcriptional programming in the MES subtype of HGSOC. Currently, only a few scRNA-seq datasets are publicly available. This becomes an even greater challenge if corresponding bulk RNA data are required for subtyping purposes. Here, we used the recently published scRNA-seq dataset by Olbrecht et al. [9] that comprised one sample of each HGSOC subtype. Although this dataset is limited in its size, it provides a valuable foundation for initial investigations related to molecular subtypes.

By applying LIANA and NicheNet, we unravelled significant ligand–receptor interactions and their effects on downstream transcription in cancer cells in the MES subtype. The composition of the TME is of critical importance for many cancer entities. Although the sample size was too scarce to make general statements about cell type abundance, with bulk xCell analysis, we attempted to characterize the TME of MES. The mesenchymal HGSOC fosters a pro-inflammatory environment comprising high quantities of stromal cell types paired with an influx of myeloid cells. We also observed a lack of other immune cell infiltrates that likewise has been reported in mesenchymal subtype tumours of triple-negative breast cancer [36]. The composition of the TME is also directly linked to the survival of patients [10]. Our analysis highlights the significance of fibroblasts in the TME and its impact on cell–cell communication. Fibroblasts have been shown to play important roles in tumour growth and progression in various cancers, including ovarian cancer [37]. They are essential drivers of intercellular signalling, providing signalling molecules such as collagens and FGFs. Of note, our analysis identified FGF18 to be expressed in both tumour cell subclusters, interacting with the FGFR1 and FGFR2 receptors of fibroblast subclusters. FGF18 has been described to be involved in para- and autocrine stimulation of tumour cells promoting cell growth and survival in several human malignancies [38]. It also drives pro-tumorigenic processes in epithelial and stromal compartments by stimulating growth and survival of tumour cells, and migration of fibroblasts, angiogenesis, and vasculogenesis [38].

The MES subtype of HGSOC is also characterized by an influx of myeloid cells. The myeloid cells of the MES sample comprised TAMs (M_CCL18), early M1 macrophages (M_CCR2), and an unspecified myeloid cell subcluster with increased CD14 expression (M_CD14). TAMs cells are strongly implicated in both the progression and chemoresistance of ovarian cancer [39]. In our analysis TAMs were the exclusive source of IL1B. Moreover, IL1B was identified as a key driver of cancer hallmarks, and its average ligand–receptor pair (IL1B–IL1R1) expression significantly correlated with single-sample enrichment scores of several cancer hallmarks in bulk MES data. Recently, IL1B has been shown to induce CXCL8 secretion in human cancer cells [40]. IL1B inhibition was able to disrupt the pathogenic cytokine loop by inhibition of pro-inflammatory factor CXCL8 [40]. The strong influence of IL1B in the TME highly suggests IL1B as a point for therapeutical intervention.

Notably, we observed a strong resemblance to the malignant cell-autonomous cytokine network we previously described [11]. Several of the key players were identified in our analysis, including the stromal cell-derived factor CXCL12, the inflammatory cytokines CXCL8, TNF, and TGFB1. We were able to elucidate the activity of several of these cytokines and their regulatory potential on hallmark genes in tumour cells.

In this study, we accumulated evidence that TGFB1 is a prominent driver of malignancy in the MES subtype, including hallmark enrichment, ligand–receptor analysis, and downstream enrichment of SMAD target genes. TGFB1 exerts stimulation on SMAD2 and SMAD3, also known as receptor-regulated effector proteins (R-SMADs), and the common mediator SMAD4 [41,42]. In turn, the TGFB signalling pathway promotes EMT and tumour malignancy [30]. In addition, TGFB1 promotes recruitment of monocytes into the TME and strongly polarizes monocyte-derived macrophages (M0) into pro-tumoral M2-like macrophages that enable tumour growth, proliferation, angiogenesis, and EMT [39,43]. Congruent to the activation of TGFB, we observed an enrichment of M2-like macrophages in the MES subtype. Importantly, M2-like macrophages are recognized to drive inflammation via TNF [39]. Furthermore, TGFB1 was proposed to contribute to immunosuppression and disease progression in ovarian cancer via the induction of TGFBI in macrophages [44].

Although we identified many similarities between the previously identified tumour promoting cytokine network and cytokine signalling in the MES subtype, IL6 in specific was neither observed in the ligand–receptor analysis nor significantly differentially expressed compared to the other subtypes [11]. Nevertheless, many studies have demonstrated the link between IL6, its transcriptional downstream activator STAT3 and EMT [9,11,45]. Increased levels of IL6 activity were observed in chronic inflammatory conditions and play a key role in growth and development in many cancers [45]. Moreover, both the significantly enriched IL6/JAK/STAT signalling hallmark and the inference of STAT3 protein activity suggest increased activity of IL6 in MES. On another note, LIF interleukin 6 family cytokine (LIF) is also a strong inducer of the JAK/STAT pathway and was significantly overexpressed in MES.

There is a complex interaction between chemokines, their receptors, growth factors, and inflammatory cytokines, often as a consequence of oncogenic mutations within the malignant cells [11,45,46,47,48]. The resultant network in the TME include activation of transcription factors such as NFKB, STAT3, and HIF1A. This complex system might be the reason why treatment of malignant cells is notoriously challenging. Hence, a particular focus of future studies may involve targeting the tumour-associated cells interrupting specific cell interactions or inducing cell–cell communication-induced apoptosis.

Many transcription factors are associated with multiple cancer hallmarks [49]. Transcription factor-induced reprogramming of cancer cells has been described as a critical aspect of tumorigenesis; these transcription factors are thus defined as oncogenes [49]. In fact, they account for about 20% of identified oncogenes in human cancers [49]. Consequently, consistently hyperactivated transcription factors such as the here-identified SMAD2/3/4, FOSL1/2, JUND, RUNX2, TWIST1, and FOXO1 might be leveraged as potential sources of therapeutic targets in the MES subtype. In fact, multiple of these transcription factors have been implicated in several tumour entities [50,51,52].

For example, the forkhead box protein O1 (FOXO1) is critically involved in the regulation of cytokine secretion and thus intercellular signalling in the TME [50]. FOXO1 has been suggested as a potential therapeutic target in oesophageal squamous cell carcinoma due to its association to the infiltration of M2 macrophages into the TME and its implication in M0-to-M2 polarization of macrophage. Remarkably, we observed an influx of myeloid cells, especially macrophage phenotypes including M1 and M2 macrophages. Similar macrophage phenotypes (M_CCL18: tumour associated macrophages; M_CCR2: early M1 macrophages) were also identified in the single-cell data [9], Ligand–receptor interaction analysis identified these macrophage subclusters as major contributors of inflammatory cytokines including members of the TNF family. Moreover, our results suggest activation of the TNF–TNFRSF1A–STAT3 axis regulating expression of key signalling molecules driving the EMT process, including TGFB1 and IL1B. Likewise, activation of the TNF–TNFRSF1A–JUN axis regulates critical ligands such as IL1B and TNF promoting the malignant microenvironment.

Finally, our study identified two novel therapeutic targets for the MES subtype of HGSOC via the identification of potential upstream alterations that propagate through signalling networks. We performed CARNIVAL analysis and identified YAP1 and NR2F6 in both the Tothill and the independent TCGA datasets.

YAP1 is an important member of the HIPPO pathway, which has a profound effect on tumorigenesis [53]. In the cytoplasm, phosphorylated and activated LATS1/2 and its adaptor protein MOB1A/B regulate cytoplasmic retention and degradation of YAP1 and TAZ [53]. Suppression of LATS1 leads to relocalization of YAP1 and TAZ to the nucleus, where they interact with the TEAD family of transcription factors. The activation of TEADs induces gene transcription that contribute to cell proliferation and survival [53]. YAP1–TEAD4 has been implicated in TIAM1-mediated activation of Rho-family GTPase RAC1, and thus, promotes tumour metastasis in breast cancer cells [54]. RAC has been linked to EMT [55]. Notably, RAC1 is also regulated by TWIST1, a highly active transcription factor in MES [55]. Similar findings have been reported in a mesenchymal subgroup of pancreatic neuroendocrine cancers with elevated YAP1 activity [56].

Among the significant ligand–receptor interactions, we identified TGF-β–VASN. Interestingly, the cell surface protein VASN has previously been implicated in the promotion of YAP1/TAZ and EMT activity in thyroid carcinomas [57].

NR2F2 and NR2F6 are members of the nuclear receptor (NR) superfamily that has been a source of therapeutic targets for the treatment of several diseases, including cancers [58]. Specifically, NR2F6 has recently gained attention as a therapeutic target boosting anti-tumour immunotherapy response [58]. The inhibition of immune checkpoint protein NR2F6 showed promising results in both pre-clinical cancer therapy in vivo and human peripheral blood mononuclear cells (PBMC) in vitro models [58]. Additionally, inhibition of NR2F6 gene function improved CD4+ and CD8+ T-cell infiltration [58]. Furthermore, synergistic benefits were observed for combination therapy comprising PD-L1 blocking in NR2F6-deficient mouse models [58]. In addition to its role in T-cells, NR2F6 has been shown to play an essential role in macrophages, as it upregulates expression of cytokines [59]. Many studies also established increased expression of NR2F6 in various cancer types including ovarian cancer, describing correlations between increased expression, faster tumour growth, and worse patient outcome [19,58,60]. However, the knowledge on the role and function of NR2F6 as a suppressor of immune response in cancer is currently limited and the fate of NR2F6-based therapy depends on the outcome of clinical trials.

Specific inhibitors for YAP1 as well as NR2F6 were previously described in the literature. For instance, CA3 was reported to inhibit YAP1 expression and transcriptional activity. Moreover, verteporfin was reported to inhibit YAP1 activity by disrupting the YAP-TEAD complex. Consequently, cell survival, cell growth, angiogenesis, and vasculogenesis were suppressed [61,62]. Like other NRs, NR2F6 shows excellent druggability [63]. A high throughput screening for compounds capable of inhibiting NR2F6 identified several small molecule compounds to the orphan NR, NR2F6 [64]. Moreover, NR2F6 overexpression has previously been described to promote the chemoresistance of epithelial ovarian cancer via activation of Notch3. It is therefore not surprising that NR2F6 expression is a biomarker for response to therapy with gamma secretase inhibitors, which inhibit Notch signalling [63]. Hence, indirect inhibition of YAP1 and NR2F6 via the HIPPO and Notch3 signalling pathways, respectively, provides intriguing alternative treatment opportunities.

Due to the critical influence of the TME on the processes promoting the malignancy, the cell lines are not adequate model systems to validate our predictions. Thus, we intend to follow-up our findings in future studies. The elucidation of appropriate model systems for in vitro or in vivo experiments is challenging. However, patient-derived xenograft (PDX) and 3D co-culture organotypic models seem promising because they recapitulate the entirety of TME.

## 5. Conclusions

In conclusion, our complementary bulk and single-cell analysis provides a deepened understanding of the TME, the cell–cell communication and its downstream effects on transcription in cancer cells of the MES subtype of HGSOC. Moreover, this study identified several consistently hyperactivated transcription factors, including FOSL1/2, JUND, NR2F2, RUNX2, and SMAD2/3, as potential sources for treatment opportunities. These candidates warrant further investigation. Likewise, YAP1 and NR2F6, two candidate therapeutic targets with potential relevance on personalized medicine and patient outcome also warrant further investigation. We hypothesize that validation in appropriate model systems will show distinct differences in cell viability based on YAP1/TAZ localization and activity. Similarly, targeting NR2F6 could lead to promising perspectives for immunotherapy regimens. Since molecular subtypes are reported for other cancer entities, approaches as described in this study could facilitate the identification of novel therapeutic strategies for a variety of tumour entities, and thus drive personalized and effective medicine.

## Data Availability

All data used in this study is publicly available.

## Supplementary Materials

The following supporting information can be downloaded at: https://www.mdpi.com/article/10.3390/cancers15123155/s1, Figure S1: Ligand activity and gene regulatory potential on MES associated hallmarks called by NicheNet; Figure S2: Correlation analysis of ligand-receptor pair expression and hallmark enrichments; Table S1: Ligand-receptor interactions called by LIANA; Table S2: Ligand activity and gene regulatory potential on MES associated hallmarks called by NicheNet; Table S3: Correlation analysis of ligand-receptor pair expression and hallmark enrichments; Table S4: Transcription factor activity inference by VIPER.
