# Supplementary figures and images for: In Silico Analysis Predicts Nuclear Factors NR2F6 and YAP1 as Mesenchymal Subtype-Specific Therapeutic Targets for Ovarian Cancer Patients
Source: Cancers (Basel). 2023 Jun 12;15(12):3155. doi: 10.3390/cancers15123155 (PMC10296561; doi:10.3390/cancers15123155)

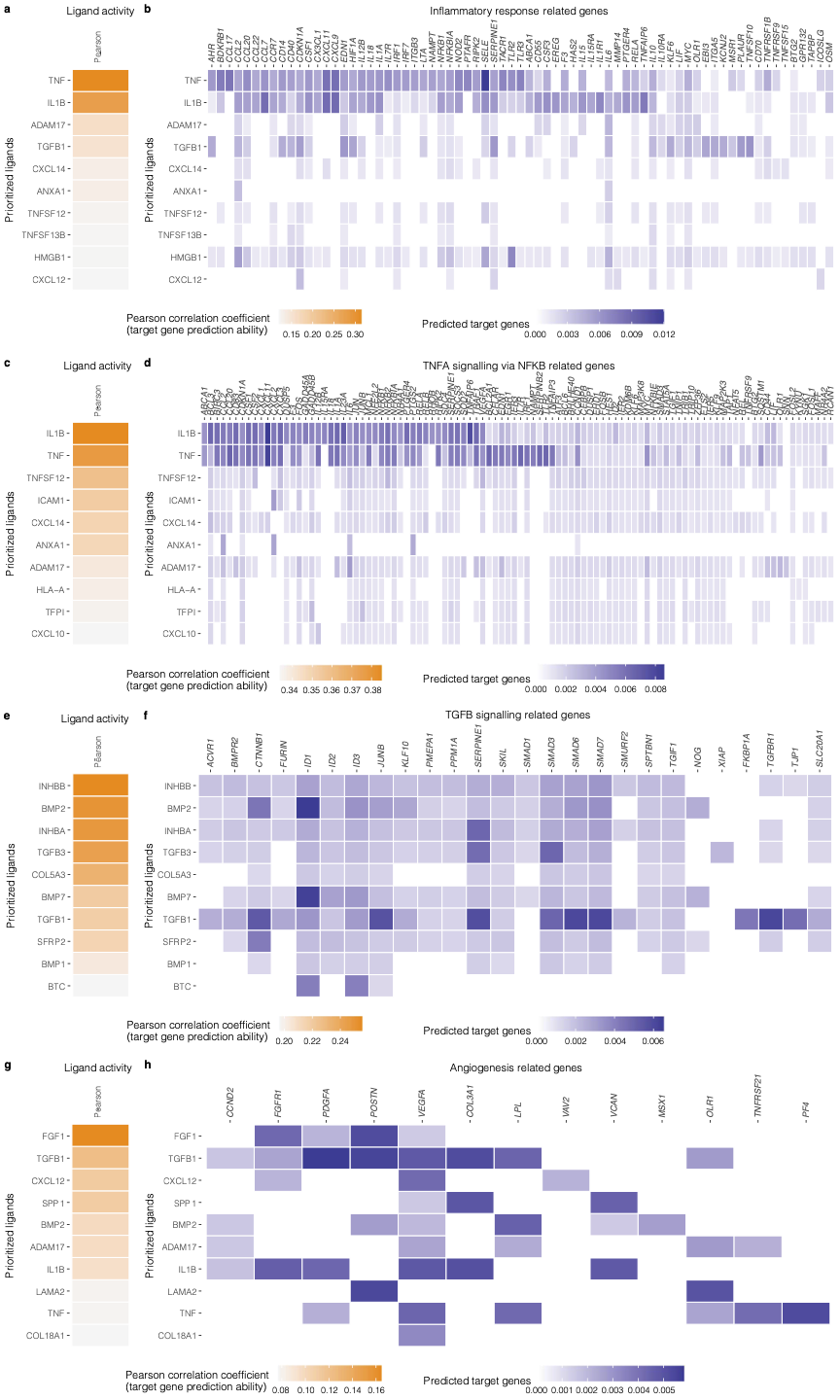

Supplement: Supplementary file 1 [file cancers-15-03155-s001.zip › Supplementary Files/Suppl.1_NicheNet_hallmark_activity_suppl.tiff]

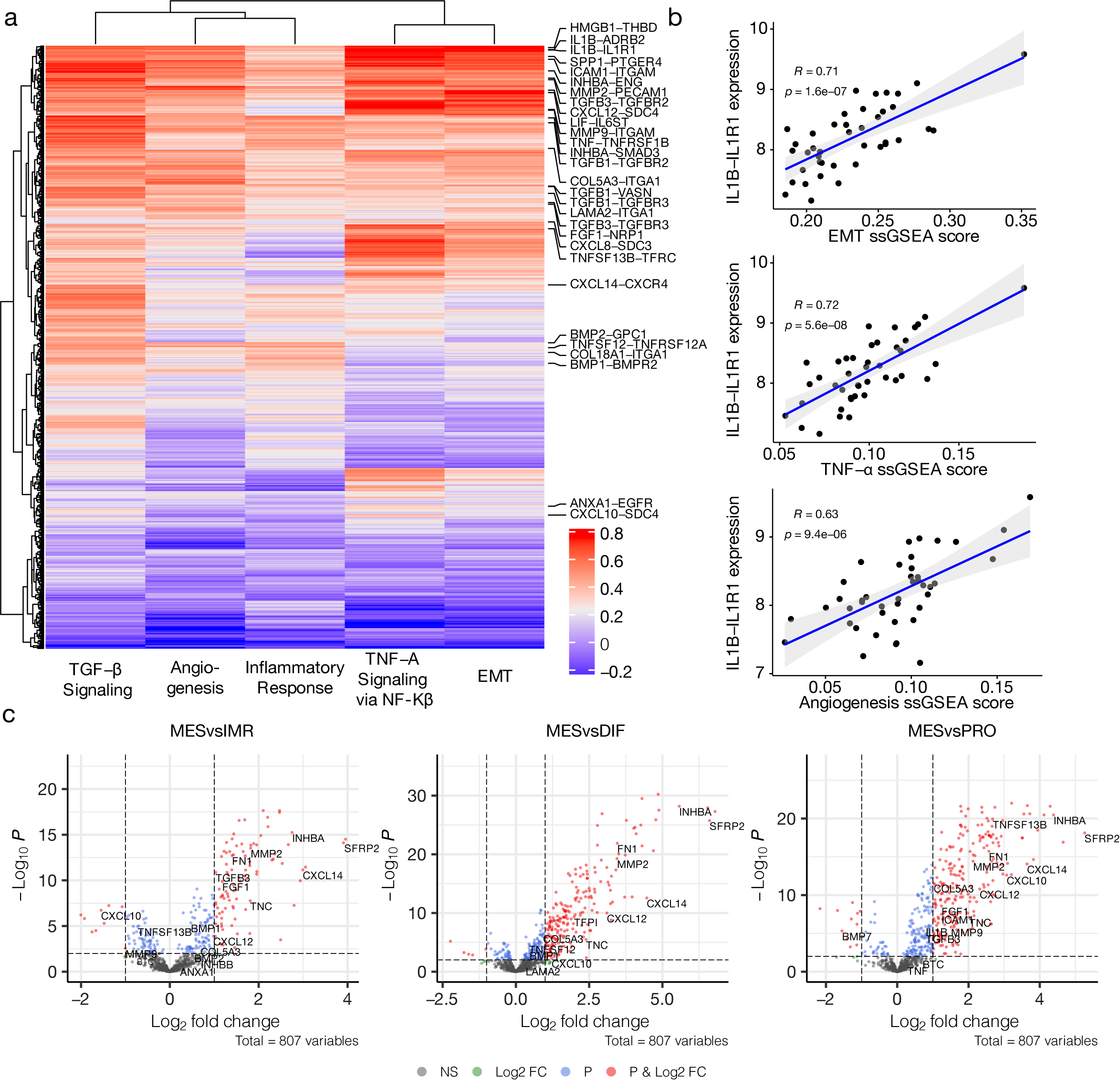

Supplement: Supplementary file 1 [file cancers-15-03155-s001.zip › Supplementary Files/Suppl.2_Enrichment correlation analysis.tif]
